# Development of anti-immunocomplex specific antibodies and non-competitive time-resolved fluorescence immunoassay for the detection of estradiol

**DOI:** 10.1007/s00216-019-01952-6

**Published:** 2019-06-08

**Authors:** Janne Leivo, Laura Kivimäki, Etvi Juntunen, Kim Pettersson, Urpo Lamminmäki

**Affiliations:** 1 Department of Biochemistry, University of Turku, Kiinanmyllynkatu 10, 20520 Turku, Finland; 2 Labmaster Oy, Fiskarsinkatu 11, 20750 Turku, Finland; 3 Inme Ltd, Itäinen pitkäkatu 4B, 20520 Turku, Finland

**Keywords:** Antibody, Immunoassay, Phage display, Antibody engineering, Estradiol

## Abstract

Detection of circulatory estradiol has widespread use in various clinical applications. Particularly, the use of estradiol-specific antibodies in immunoassays is routinely used, mainly due to the cost efficiency and simplicity of the sample handling process. However, the circulatory levels of estradiol can be extremely low in some conditions, and beyond the current detection limit of existing competitive immunoassays. We describe the generation of anti-immunocomplex specific antibodies derived from synthetic antibody repertoire and the development of high-performance non-competitive immunoassay for the detection of estradiol. Phage display selections were used to isolate new antibodies from synthetic antibody library with the use of existing estradiol specific Fab fragment. The found antibodies were consecutively used to set up a time-resolved fluorescence-based immunoassay (TRFIA), which can be used to detect estradiol with exceptional sensitivity and specificity. The limit of detection and EC50 were shown to be 3.0 pg mL^−1^ and 32.4 pg mL^−1^ respectively.

**Figure:**
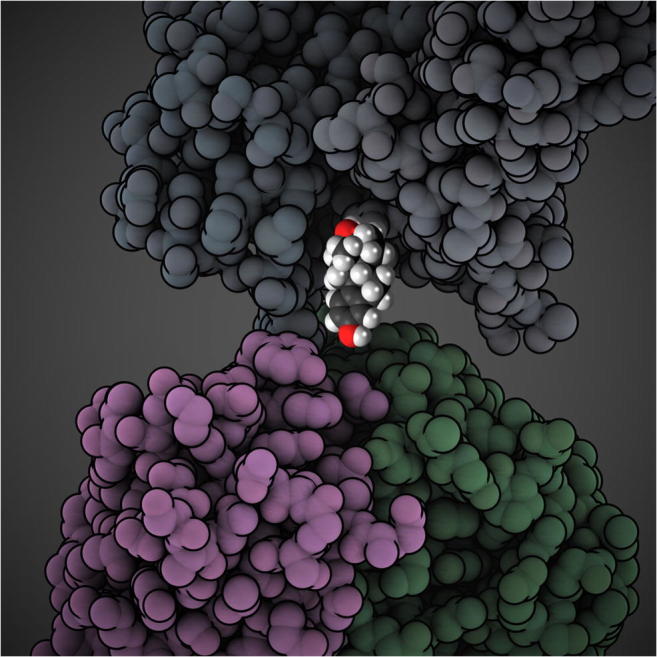
Graphical abstract

Graphical abstract

## Introduction

Monitoring the levels of estrogenic hormones in circulation can provide pleiotropic information, useful in the evaluation of gonadal functionality related to infertility, menopausal state or male feminization. Particular interest in clinical diagnostics and research has gained the measurement of estradiol (E2), the most potent of the naturally occurring estrogenic hormones [1]. The accurate measurement of E2 in clinical setting is hampered by the very broad range of circulatory levels, ranging from < 20 pg mL^−1^ in prepubertal children to > 10,000 pg mL^−1^ during pregnancy and ovarian stimulation treatment [2]. Furthermore, the clinically relevant information extracted from the circulatory E2 levels for example during aromatase inhibitor therapy for breast cancer requires high-performance detection methods with adequate sensitivity [3]. Besides sensitivity, the routine measurement of E2 often requires rapid and economical solutions to process large samples quantities efficiently.

The most established method for the measurement of E2 from biofluids is based on chromatographic separation of E2, often combined with mass-spectrometric (MS) analysis [4]. More recently, technological advancements have simplified this analytical process; particularly, the combination of HPLC and tandem MS has gained increasing focus [5, 6]. Such methods which are based on the physical separation of the target analyte require specialized instrumentation and are not suitable for high-throughput screening. Furthermore, the possibility to measure E2 levels from unprocessed biofluids in a more simple, decentralized manner is often preferred.

Immunoassays for the measurement of E2 have been in routine clinical use since the late 1960s [7]. Although the immunoassays for the measurement of circulatory E2 have evolved both in terms of detection limit and specificity, the issues related to the poor sensitivity and reproducibility make existing assays invalid for epidemiologic studies [8, 9]. Majority of the commercially available immunoassays for the detection of E2 are based on competition of the free and labelled E2. As the sensitivity of the competitive assays is intrinsically dependent on the amount of available binding sites, these assays are more prone to interferences caused by matrix effects and cross-reactive compounds. In addition, the sensitivity of competitive immunoassays is often lower in comparison with the non-competitive equivalent and is unsuitable for the measurement of samples containing low E2 levels (< 40 pg mL^−1^). Although the benefits of using non-competitive format to set up new assays for E2 detection are evident, the development of such assays is challenging due to the extremely small size of the analyte. For example, the traditional sandwich-type immunoassay, which utilizes the binding of two different antibodies against the same target, is not possible due to the limited amount of available epitopes on E2. This limitation can be circumvented in some cases with the use of immunocomplex specific antibodies [10]. There has been a steady increase in studies describing the use of immunocomplex antibodies for the detection of low-molecular-weight compounds [11, 12].

One of the main limitations for more widespread use of anti-immunocomplex specific antibodies is the tedious antibody development process, which can be very challenging with traditional methods based on the immunization of animals [13]. With the introduction of in vitro antibody development methods, the selection conditions throughout the antibody development process can be controlled more efficiently enabling the generation of antibodies with predefined specificity to complex targets. Phage display has been used to create immunocomplex specific binders against small analytes such as morphine [14], mycotoxins [15], and cyanotoxins [11]. In comparison with conventional competitive immunoassays, the use of immunocomplex assays has multiple benefits. (1) Since the interaction is based on the binding of the two antibodies, there is no need for a chemically conjugated or labelled analyte for the detection. (2) The intrinsically non-competitive format results in enhanced binding kinetics, assay sensitivity, and linear range. (3) Versatility on the design of assay concept results from the use of two antibodies in the assay setup. In addition to the use in conventional non-competitive ELISA, immunocomplex-based assays have been successfully implemented in homogenous formats utilizing advanced reporter technologies such as TR-FRET [12].

**Fig. 1:**
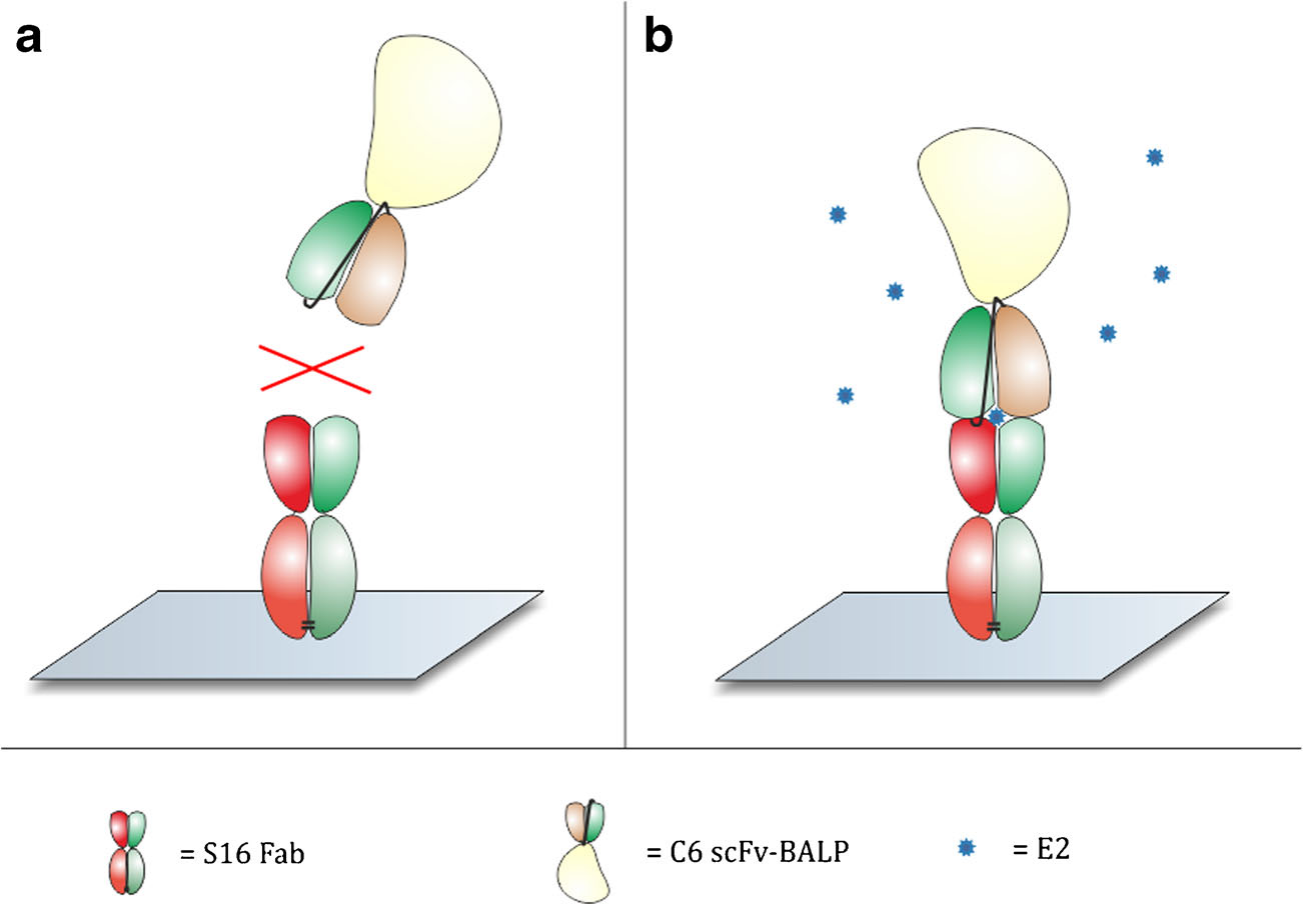
The concept of an immunocomplex assay. **a** In the absence of free E2, the C6 scFv-BALP antibody does not interact with the immobilized S16-Fab. **b** The formation of immunocomplex is dependent on the interaction of E2 to the binding site of the S16 Fab

We describe the generation of anti-immunocomplex specific antibodies derived from synthetic antibody library. The found antibodies were utilized in the development of a non-competitive immunocomplex immunoassay which can detect E2 with high sensitivity. The detection is based on the binding of the secondary antibody in the presence of both primary S16-Fab and soluble E2 (Fig. 1). The half-maximal effective concentration (EC50) and limit of detection (LoD) were 32.4 pg mL^−1^ and 3.0 pg mL^−1^ respectively, indicating sufficient performance for the measurement of samples containing very low levels of circulatory E2.

## Materials and methods

### Materials and reagents

For the immunoassays, DELFIA series buffers and streptavidin-coated 96-well microtiter plates were purchased from Kaivogen Diagnostics (Turku, Finland). All measurements were done with Victor 1420 fluorometer from Perkin-Elmer (Turku, Finland). Magnetic nanoparticles and magnetic bead concentrator and hyperphages were from Invitrogen (Thermo, USA). The hormones used in this study, 17β-estradiol estrone, estriol, and 17β-estradiol-3-glucoronide, were from Sigma-Aldrich (USA). The *E. coli* cell lines used for the sorting and expression of the antibody libraries were purchased from Stratagene (USA): BL21 (F-, dcm, ompT, hsdS [rB- mB-], gal [malB+], K-12[λS]) and XL1-Blue (recA1, endA1, gyrA96, thi-1, hsdR17, relA1, lac [F′, TetR]). All microbiological reagents were prepared as described in Sambrook et al. [16]. The single-chain alkaline phosphatase (scFv-BALP) fusion proteins were purified with Ni-NTA (Thermo Scientific, USA). The ELISA substrate para-nitrophenylphosphate (pNPP) and activated charcoal used for the depletion of hormones from the serum samples were obtained from Sigma-Aldrich (USA).

### Standard solutions and samples

The E2 standard solutions were done in absolute ethanol (EtOH). The primary stock was done by dissolving E2 in EtOH in the concentration of 20 mM. For the assays, the working concentrations were established in the range of 0.1–10,000 pM. For the analysis of sample matrix interference, the standard concentrations were set in the range of 6.25–150 pM. The serum samples used to study the matrix effects were obtained from healthy young male volunteers.

### Antibody development

Previously well characterized anti-E2 S16 Fab was used for the capture of E2 in both phage display selections and immunoassays [17]. For the development of the secondary antibody specific to the immunocomplex, synthetic antibody libraries and phage display were used, described in previous studies by Brockmann et al. [18] and Huovinen et al. [19]. The pEB32x phagemid vector used for the phage display selections was designed to contain a trypsin cut-site between the antibody fragment and the truncated pVIII phage coat protein. The phage display selections were carried out with following conditions: S16 Fab (8 μg) which was covalently coupled to Tosyl-activated paramagnetic beads (Dynal, Norway) supplemented with E2 (10 μM) for 1 h in rotation. The beads were washed three times with TBT-0.1 (50 mM Tris, 150 mM NaCl, 1% BSA-fraction V, 0.1% Tween-20, pH 7.5) buffer and 1 × 10^12^ tfu of library phages was mixed with beads. The step was supplemented with unspecific mouse IgG (100 μg) to deplete the antibodies recognizing regions not involved in the antigen binding. The solution was incubated for 2 h at room temperature (RT). The beads were washed three times with TBT-0.1 buffer and once with TSAT (50 mM Tris, 150 mM NaCl, 0.05% Tween-20, pH 7.5) before elution with 10 μg/ml of trypsin for 30 min at RT. The eluate was used to infect XL1-Blue cells in exponential growth phase. The phages were repropagated from the cells collected from the output plate as described previously. For the second and third rounds of phage display selections, the beads were changed to Dynabeads M280 (0.1 mg) which were coated with biotinylated-S16 Fab (bio-S16 Fab). The amounts of S16 Fab, E2, and phages were reduced after each selections round to increase the selection stringency.

#### Antibody screening and characterization

For the screening, the antibody gene pool from the third selection round was cloned to pLK06H expression vector to enable the production of the scFv fragment in fusion with bacterial alkaline phosphatase (BALP). Individual colonies (*n* = 190) were screened from the transformation plate and cultured on a 96-well tissue microtiter plate in 150 μL volume of SB supplemented with 100 μg/ml of ampicillin, 10 μg/ml tetracycline, and 0.05% glucose. The scFv-BALP production was induced with 250 μM IPTG. The overnight production of the scFv-BALP was done in plate shaker with 900 rpm at + 26 °C. The cells were removed with 6800*g* centrifugation for 30 min at + 4 °C and 10 μL of the culture supernatant was used in the primary screening immunoassay. For the more detailed characterization, the antibodies were produced in 400 ml culture of *Bl21* cells and consecutively purified as described previously [20].

### Immunoassays

Phage preparations isolated after each selection round were used in an immunoreactivity assay, where the enrichment of the immunocomplex specific phages was monitored with the use of TRF immunoassay using the following conditions. The S16-Fab (100 ng) was immobilized on streptavidin-coated microtiter wells for 1 h. After four washes, E2 (50 nM) and repropagated phages from each selection round (1 × 10^8^ tfu) were added to the wells and incubated for 2 h. The wells were washed twice before the addition of europium-labelled anti-fd IgG.

The screening of the scFv-BALP antibodies was done in two phases: the primary screening was done with single colony screening from the culture supernatant of the 96-well plate. The screening assay was used to determine the signal differences from two wells containing the S16-Fab (100 ng) with and without E2 (50 nM). The most promising clones were selected for further characterization in secondary screening assay, where 100-ng bio-S16 Fab was used to capture E2 with five different concentrations (10, 30, 100, 300, 1000 pM).

The selectivity of the parental S16-Fab has been previously shown to be very specific to E2 with low cross-reactivity for other estrogenic compounds. To confirm this, we analyzed the cross-reactivity of the assay against three estrogens: estrone (E1), estriol (E3), and estradiol-3-glucoronide (E2-gluc), which were added in three concentration (15, 50, and 100 nM) to the immunoassay.

After optimization steps, the assay condition was set as follows: the streptavidin wells were coated with 5 ng of bio-S16 Fab and incubated for 1 h. After four washes, serial dilutions, ranging from 0.1–10,000 pg L^−1^, of E2 were added to the wells as triplicates for each concentration point. For detection, C6 scFv-BALP antibody (10 ng) and Eu-labelled anti-BALP antibody (15 ng) were added to each well and the plates were incubated for 2 h. The wells were washed four times before the addition of DELFIA enhancement solution. After 15-min incubation, the TRF europium signal was measured.

To study the matrix effect of serum in the immunocomplex assay, serum was collected from three healthy male donors with written consent from the lab members of the Biotechnology Division, University of Turku. The hormones were stripped from the serum with the use of 0.1% activated charcoal. The charcoal was removed from the samples with centrifugation at 132,000*g* for 15 min, followed by a filtration with 0.22-μm filter. For the immunoassay, variable amounts of E2 ranging between 6.25 and 150 pM were spiked to samples to analyze the matrix effect. A 1:10 dilution of the serum sample was used for the determination of the recovery capability of the immunoassay.

### Data management

The graphs for the assay performance and determination of the EC50 values and *R*^2^ were done with the use of GraphPad Prism (GraphPad Software Inc.). The general analytical parameters LoD and LoQ were calculated from the mean of the background + 3 × SD (LoD) and 10 × SD (LoQ). The intra- and interassay imprecision was determined from the mean of CV% with the use of E2 spiked in three concentrations (25, 50, 100 pM) to pooled serum samples (*n* = 3), which were analyzed in two consecutive days.

## Results and discussion

### Antibody development and characterization

**Fig. 2:**
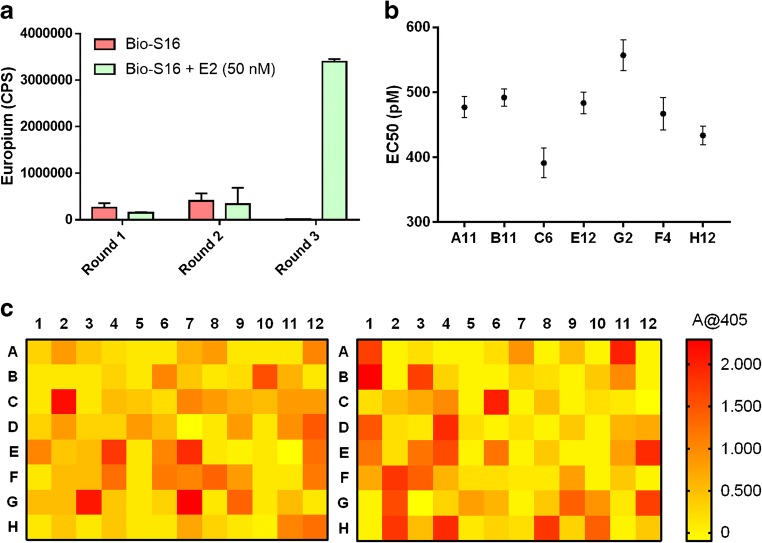
**a** The primary screening for the immunocomplex antibodies was done in 96-well plates where the binding of the clones was distinguished between signal differences of two wells containing the S16-Fab with and without E2 (50 nM). The heatmap shows the specific signal of absorbance measured at 405 nm. **b** Seven of the most promising clones were selected for further characterization with an ELISA consisting of five concentration points of E2, the EC50 values; the error bars represent the standard error of the EC50. **c** The immunoreactivity of the phage stocks after each phage display selection round. The error bars represent the standard deviation of three replicate measurements

In this study, we used previously established synthetic antibody libraries, and a high-affinity recombinant Fab fragment specific to E2 as a source of immunocomplex specific binders. The synthetic antibody libraries were constructed on the basis of phage display selections, which provides greater control over the various parameters of the antibody selection process in comparison with immunization-based antibody generation strategies. To direct the phage display selection pressure towards the antigen-binding regions of S16-Fab complexed with E2, we used both intact mouse IgG and unconjugated S16-Fab to deplete the unspecific binders prior to the introduction of the target immunocomplex. In addition, the overall stringency of the selections was increased throughout the selection rounds, both with the use of additional washing cycles and with the reduction of the amount of S16-Fab and E2 used for the immobilization. Based on the number of colonies on the output plates obtained after each selection round, and the immunoreactivity assay done to the phage stocks, a strong immunocomplex specific enrichment was observed after three selection rounds in the presence of 50 nM of E2 (Fig. 2a). In the third round phage pool, there was no detectable binding against the S16-Fab without soluble E2 in the immunoreactivity TRFIA, indicating a successful depletion of the phages specific to the regions not involved in the binding of E2.

The primary single colony screening of individual scFv-BALP antibodies was done with conventional ELISA utilizing the catalytic activity of the BALP in the presence of pNPP. From the 190 colonies screened, 26 clones were above the threshold set for the assay (signal/background level > 5), and seven were selected for further characterization (Fig. 2b, c). Based on the secondary screening done with immunoassay consisting of increasing E2 concentrations, all seven clones were specific for the S16 Fab-E2 complex with EC50 values below 1 nM (Fig. 2b). The most promising clone with lowest EC50 values, C6 scFv-BALP, was produced in larger quantities and subjected to further characterization and assay development.

### TRF immunoassay development and optimization

**Fig. 3:**
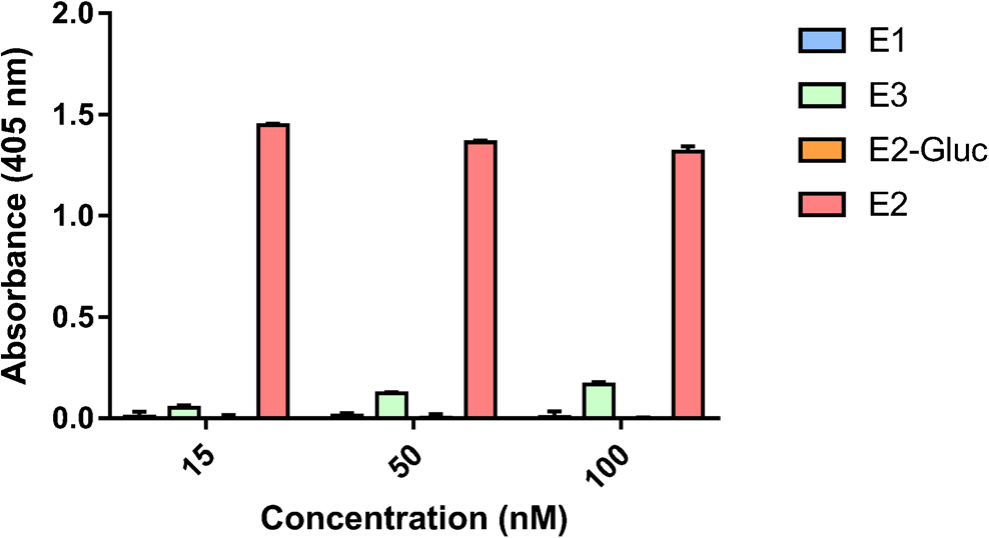
Selectivity of the immunoassay as analyzed with the use of four different estrogens (E1, E3 E2-gluc, and E2). Each estrogen was added in three concentrations (25, 50, and 100 nM). The error bars show the standard deviation of three replicate measurements

The binding characteristics of the S16-Fab were previously shown to bind E2 with high affinity (0.27 nM) and specificity, establishing a good starting point for the development of a non-competitive immunocomplex assay [17]. The selectivity of the found C6 scFv was analyzed with the use of different estrogens (E1, E3, and E2-gluc) in comparison with E2 (Fig. 3). No observable reactivity could be detected for estrone. For the structurally similar E2-3-glucuronide, which has caused cross-reactivity issues to the parental E2-specific antibodies, the assay did not show any reactivity. The only compound tested which caused interference in the assay was estriol especially at higher concentration (> 100 nM). However, for clinical use, this most likely will not be an issue considering the very low levels of circulatory E3 in non-pregnant women. Moreover, the selectivity of the assay clearly was towards E2, as the EC50 values could not be determined for none of the other estrogenic compounds.

Prior to the full characterization of the C6 scFv-BALP binding properties, the antibody was produced in a larger scale and purified. The assay optimization was started by adjusting the general parameters, such as incubation time, buffer pH, antibody concentrations and amount of washes, prior to the analysis of final assay performance (results not shown). Although the initial screening and characterization of the scFv-BALP antibodies were done in ELISA format, the TRFIA-based reporter was used in the final assay setup for enhanced sensitivity.

### Assay performance

**Fig. 4:**
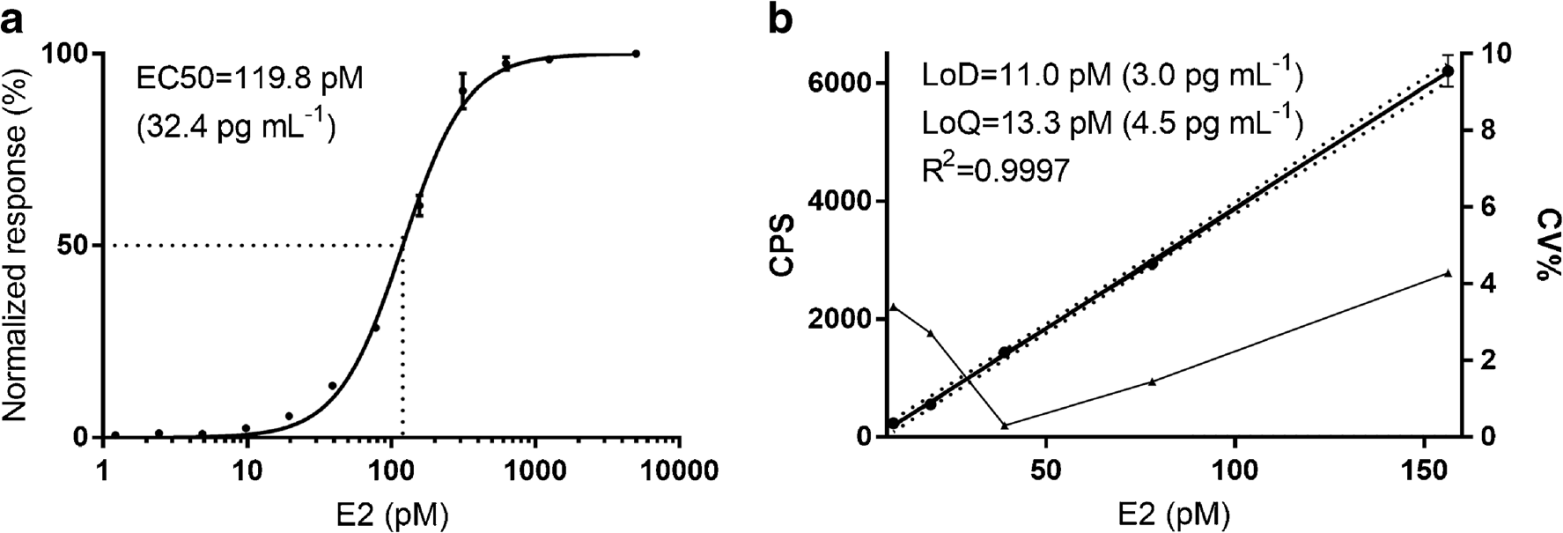
Assay performance with optimized conditions. **a** Standard curve for the detection was done on the basis of 12 concentration points of E2 ranging between 0.1 and 10,000 pg L-1. The error bars indicate the SD of three replicate measurements. **b** Limit of detection and limit of quantification were calculated from the mean of the background + 3 × SD and 10 × SD respectively

For the determination of half-maximal effective concentration (EC50), 12 concentration points of E2 ranging between 0.1 and 10,000 pg L^−1^ were used (Fig. 4). The EC50 for optimized assay was found to be 119 pM (32.4 pg mL^−1^), with a limit of detection (LoD) of 11 pM (3.0 pg mL^−1^) and limit of quantification (LoQ) of 13.3 pM (4.5 pg mL^−1^). These values suggest promise for the detection of low-range concentrations of E2, which is currently the most problematic sample group for existing E2 assays [21, 22].

**Fig. 5:**
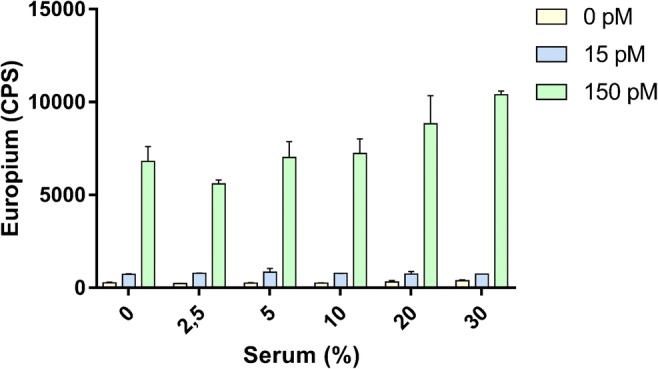
Matrix effect on the TRFIA. The effect of increasing amounts of serum, ranging between 2.5 and 30%, on the assay was assessed using two concentration points of E2 (15 and 150 pM). The error bars represent the SD of the europium signal

The optimal conditions for the final immunocomplex assay were used to characterize the more specific parameters of the assay. First, we determined the significance of the matrix effect on the antibody interaction with the use of charcoal-stripped serum samples pooled from three donors. The addition of serum to the immunoassay had only minor effect on the signal levels of the assay as shown in Fig. 5. For the matrix effect tests, the serum was stripped of free hormones to reduce the effect of soluble E2. The removal of E2 clearly was not complete as can be observed in Fig. 5, where a slight, but significant, increase in signal can be observed with the increasing amount of serum added to the well. The overall interference caused by the use of serum as a sample matrix was minimal with acceptable variability. Next, we spiked variable amounts of E2 to three individual charcoal-stripped serum samples obtained from healthy volunteers (Table 1). The recoveries ranged between 82 and 167% with acceptable CV%. Higher recoveries were observed in lower E2 concentration, which is a well-known source for error when approaching the detection limit of the immunoassay. Next, the imprecision of the assay was evaluated by analyzing three pools of serum samples in triplicate in two consecutive days (Table 2). Overall, the imprecision remained acceptable along the different concentration points of E2 measured. Apart from the lowest E2 concentration (25 pM), the imprecision of the system remained below 10%. Although the assay described in this study was optimized for the detection of E2 from serum samples, similar approach can be used in the future for the analysis of E2 from less complex matrices, such as urine and waste water.

**Table 1:** Recovery of E2 from spiked serum samples. Three charcoal-stripped serum samples were used to assess the recovery capability of the E2 immunoassay. The serum samples were spiked with five concentrations of E2 in three different healthy males

Spiked (pM)	Serum					
	S1		S2		S3	
	Found	CV%	Found	CV%	Found	CV%
6.25	9.5	1.5	8.4	6.4	10.5	8.5
12.5	15.1	0.7	14.7	0.4	15.6	2.2
25	25.3	2.0	27.3	5.5	25.7	4.1
50	42.8	9.6	52.3	1.9	43.6	4.5
100	92.5	8.4	105.8	14.2	81.7	3.6

**Table 2:** Intraassay, interassay, and total imprecision of the immunoassay in pooled serum samples at three E2 concentrations

E2 concentration (pM)	CV%		
	Intraassay (*n* = 3)	Interassay (*n* = 3 × 2)	Total
25	6.7	4.9	11.5
50	2.6	4.0	6.6
100	4.1	4.4	8.5

Immunocomplex-based assays against E2 and other small molecules enable the development of versatile assay setups for the detection and screening of the respective compounds. The non-competitive assay format overperforms the competitive counterparts in the most crucial aspects of a reliable immunoassay. In addition, the described assay setup which consists of two recombinant antibodies can be easily converted into homogeneous format utilizing advanced reporter technologies such as TR-FRET [23] and upconverting phosphors (UCP) [24]. Most widely used application for the detection of E2 is mainly done in the clinic; during the assessment of gonadal activity, applications for E2 monitoring can be found also in environmental and veterinary diagnostics [25, 26]. Although the assay described in this study needs to be validated with a larger sample set, where the E2 values have been established with a reference method, our assay demonstrates potential in the detection of very low E2 concentrations from unextracted serum samples. The assay demonstrates high performance in terms of analytical sensitivity, and with further optimization or engineering the binding properties of the C6-scFv antibody, it is possible to establish new tools for the detection of E2 concentrations ranging between 0.2 and 2 pg mL^−1^, which currently is lacking reliable and accurate methods.
